# Serous tubal intraepithelial carcinoma—looking into the earliest events of ovarian cancer development

**DOI:** 10.20935/acadonco7620

**Published:** 2025-03-25

**Authors:** Ie-Ming Shih, Yeh Wang, Russell Vang

**Affiliations:** 1 Departments of Pathology, Gynecology and Obstetrics, Johns Hopkins Medical Institutions, Baltimore, MD 21231, USA.

**Keywords:** ovarian high-grade serous carcinoma, serous tubal intraepithelial carcinoma, p53 mutation, BRCA mutation, CCNE1 and MYC amplification, aneuploidy

## Abstract

Among human malignancies, ovarian epithelial neoplasms are unique because they are unlikely to develop from their cognate organs, the ovaries (specifically, the surface mesothelium). The updated paradigm about the origin of high-grade serous carcinoma (HGSC) suggests that many HGSCs derive from the fallopian tubes following a sequential tumor progression, from pathologically defined p53 signature, serous tubal intraepithelial lesion, and serous tubal intraepithelial carcinoma (STIC) to HGSC that later spreads to ovarian tissues and disseminates. Despite the fact that the biological and clinical significance of each of those precursor lesions is yet to be elucidated, molecular and morphological correlative studies demonstrate unique features associated with various precancerous lesions. Chromosomal instability, aneuploidy patterns, and the activation of specific cancer signaling pathways attribute tumor progression to HGSC. The knowledge gained thus far is transforming various aspects of ovarian cancer research and gynecological practice. Opportunistic salpingectomy prevents HGSC in average-risk women, and molecular analyses in routine liquid-based cervical Pap tests hold promise to detect STIC- and HGSC-related tissue biomarkers. This review article will summarize those key findings in the earliest development of HGSC precursors and discuss the future challenges and promises of translating this paradigm shift to leverage standardization in diagnostic, early detection, and prevention of this devastating ovarian cancer.

## 1. Introduction

Ovarian epithelial cancer can be broadly classified into Type I and Type II neoplasms based on their distinctive histological features and unique molecular genetic alterations [1]. Type I ovarian cancer includes low-grade serous, endometrioid, clear cell, and mucinous types, whereas Type II ovarian cancer encompasses high-grade serous, carcinosarcoma, and undifferentiated types. A number of features distinguish Type II ovarian cancers from Type I neoplasms [1]. Type II ovarian cancers are more frequently high-stage at diagnosis, and they harbor TP53 mutations in almost all tissue samples tested [2]. Approximately half of Type II cancers have defective homologous recombination DNA repair pathways, and the other half show the amplification or upregulation of *CCNE1*. In contrast, Type I ovarian tumors have more abnormalities in genes involving mismatch DNA repair and the ARID1A, PI3K, KRAS/BRAF, Wnt, and protein phosphatase 2A pathways. High-grade serous carcinoma (HGSC) is the most common subtype of ovarian cancer and is the primary focus of the following discussion.

HGSC is commonly detected in advanced stages when clinical interventions are less effective. Therefore, early diagnosis and prevention targeting the precursor stages or early-stage diseases offers the best hope of reducing the morbidity and mortality of HGSC. The scientific premise for early cancer diagnosis and prevention lies in a better understanding of the molecular alterations that contribute to tumor initiation and progression.

While the tissue origin of several Type I ovarian cancers, like clear cell and endometrioid carcinomas, has become associated with ovarian endometriotic cysts, the origin of serous types of ovarian cancer was largely unknown until attention shifted to the fallopian tubes. This review will discuss the updated paradigm of ovarian HGSC development and summarize the recent studies that shine new light onto the earliest events in ovarian cancer precursor lesions.

## 2. The tubal paradigm in the development of high-grade serous carcinoma

**Figure 1 •:**
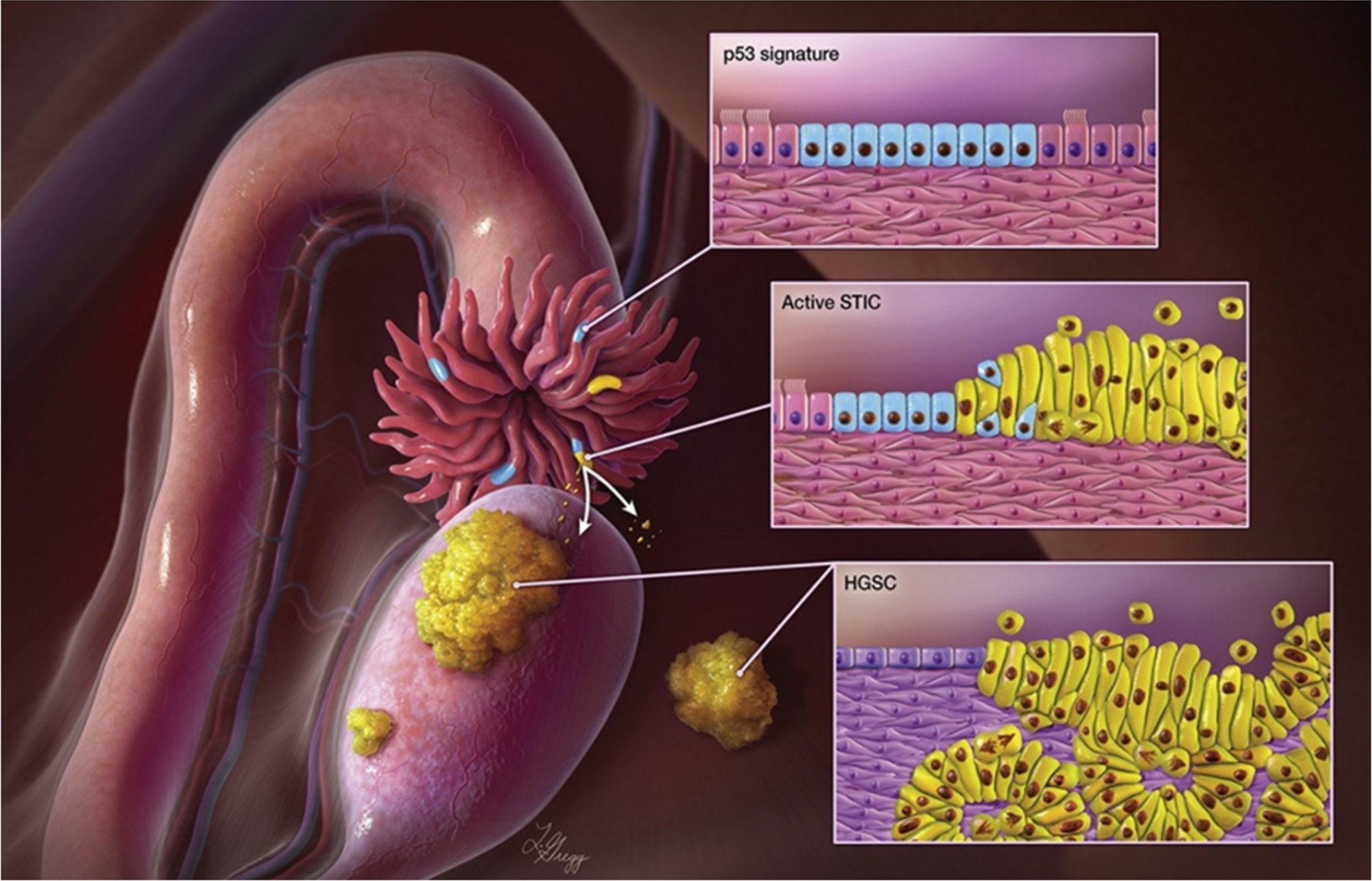
Tubal paradigm in the origination of high-grade serous carcinomas (HGSCs). The fimbriated end can be affected by a variety of fallopian tube lesions, including serous tubal intraepithelial carcinoma (STIC) (yellow lines) and p53 signature (blue lines). STIC is thought to be an immediate predecessor of HGSC. STIC cells can invade the fallopian tube and detach from its surface, spreading to the peritoneal surface and enveloping the ovary, bowel, peritoneal wall, and omentum. Natural selection favors emigrated STIC cells that can survive and multiply in a specific tissue-environmental niche, resulting in tumor nodules and ascites. This illustration was authored by Ie-Ming Shih and depicted by Lydia Gregg, Copyright 2020 I. Shih JHU; used with permission by Dr. Shih.

High-grade serous carcinoma has traditionally been considered to originate de novo from the ovaries [3, 4]. Nonetheless, insufficient evidence raises a concern about the validity of this hypothesis and instead suggests that HGSC precursors originate from somewhere else. Approximately twenty-four years ago, a European study utilizing meticulous histopathological analysis of adnexal tissues from women with *BRCA1* germline mutations identified dysplastic epithelial cells indicative of high-grade serous carcinoma precursor lesions in the fallopian tubes rather than on the ovarian surface [5–7]. Subsequently, several research teams independently provided evidence that ovarian HGSCs appear to emerge from microscopic precursor lesions of the fallopian tubes [8–10]. HGSC precursor lesions are now known as serous tubal intraepithelial carcinomas (STICs). Later on, abundant data from clinicopathological investigations, clonal trajectories derived from molecular genetic analyses, gene expression, and methylation profiles, as well as population-based studies of salpingectomy, corroborated a fallopian tube origin for the majority of ovarian HGSCs (Figure 1) [8, 10–25].

For example, population-based studies provide cogent evidence to support the tubal paradigm. At least three such studies are reporting a decrease in the occurrence of ovarian cancer among women who have undergone salpingectomy for different causes [23–27]. A study conducted in Sweden found that the risk of ovarian cancer fell by 29% in the group that underwent unilateral salpingectomy and by 65% in the group that underwent bilateral salpingectomy [27]. By examining a population-based dataset that covered 23 million people countrywide in Taiwan, investigators identified a 30% reduction in women with ectopic pregnancy who underwent unilateral salpingectomy [23]. Women in the Canadian Province of British Columbia who underwent opportunistic bilateral salpingectomy during hysterectomy for benign reasons or as an alternative to bilateral tubal ligation did not have any instances of HGSC in contrast to the projected number of 8.68 cases in the age-adjusted control group [26].

STIC is microscopically defined by nuclear atypia and architectural abnormalities in epithelial cells, together with sporadic mitoses and occasional apoptotic bodies. The proliferative activity in STICs, shown by the Ki-67 labeling index, typically exceeds 10% [16, 28, 29]. In addition to STIC, another associated lesion is identified as a “serous tubal intraepithelial lesion” or STIL. In contrast to typical STICs, STILs exhibit milder nuclear and architectural anomalies. Their proliferative activity may be comparable to or marginally elevated relative to the neighboring normal-appearing fallopian tube epithelium. It is important to highlight that incidental STICs are rare, and the interobserver reproducibility in diagnosing STIC remains not high, even among experienced gynecologic pathologists [17].

Consequently, some researchers designate STIL as proliferatively dormant STIC. A prevalent perspective suggests that STIL likely serves as a precursor to STIC. The transformation from an STIL to an STIC entails supplementary molecular genetic or epigenetic modifications that facilitate their proliferation. Conversely, STILs encounter a transformative impasse as the lesion ceases to advance due to oncogene-induced senescence or growth arrest.

**Figure 2 •:**
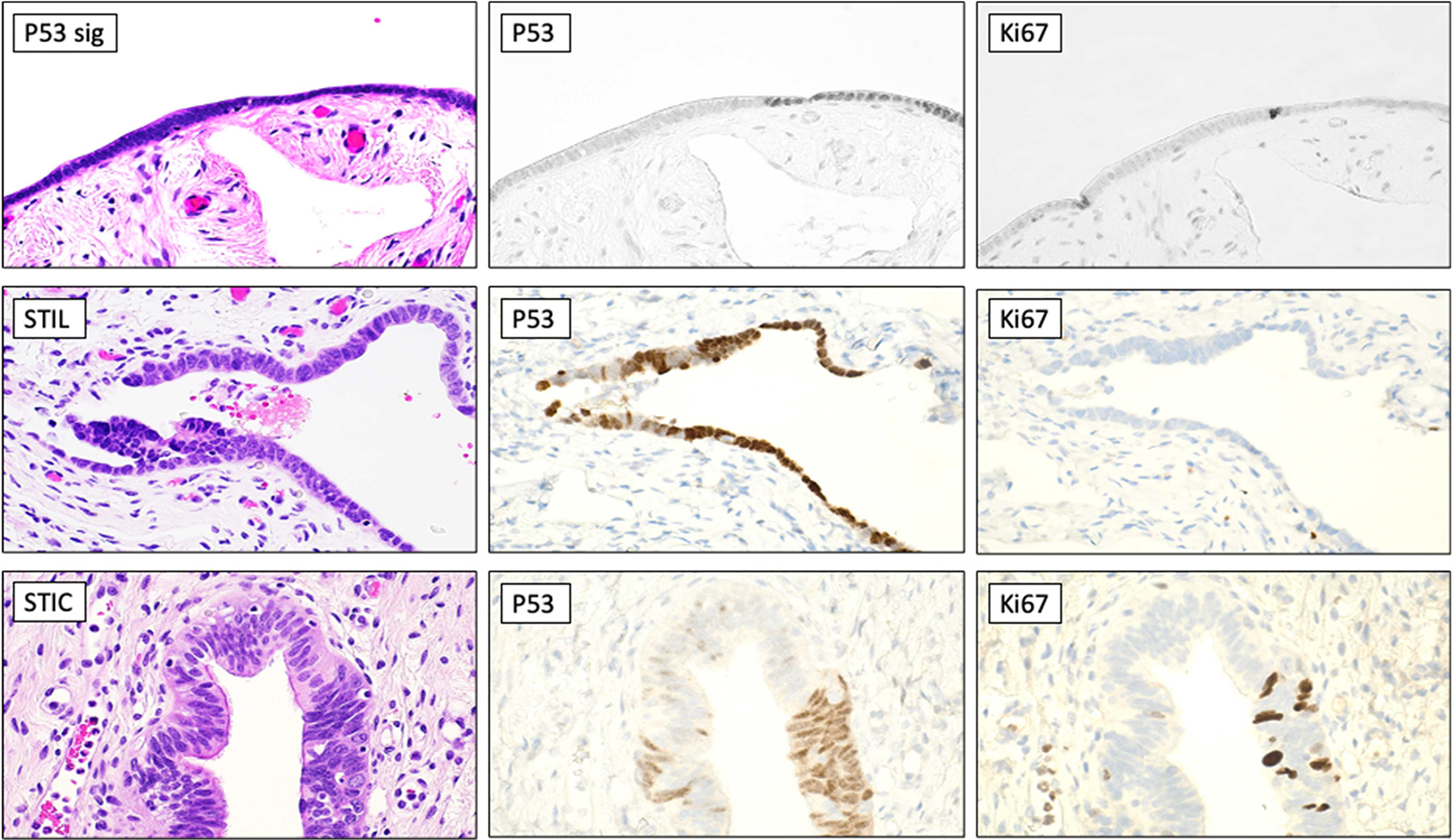
Examples of tubal precursor lesions of high-grade serous carcinoma. Top row, representative H&E, p53, and Ki-67 stains of a p53 signature. Middle row, representative H&E, P53, and Ki67 stains of an STIL. Bottom row, representative H&E, p53, and Ki-67 stains of an STIC. The upper panels were taken at 10x and bottom panels at 40x.

Molecular alterations may occur prior to detectable morphological changes identified by pathologists. During the application of p53 immunohistochemistry to study STICs and STILs, investigators identify a minute stretch of histologically unremarkable fallopian tube epithelial cells labeled with intense p53 immunoreactivity in a contiguous fashion. Those stretches of cells were analyzed to show missense *TP53* mutations. Accordingly, pathologists call them “p53 signatures” [8, 30]. The biological and clinical relevance of p53 signatures remains uncertain; nonetheless, they are likely to be indolent and accidental observations. It remains uncertain whether STILs and p53 signatures will be “waken up” to regain momentum in proliferation and manifest themselves with more genetic variations for selection to become STICs. Therefore, the evolvability of STILs and p53 signatures awaits further studies. Representative images of H&E and immunohistochemistry stains of these tubal precursors are shown in Figure 2.

In contrast to other carcinoma types that confront anatomical obstacles (such as the submucosa, muscular layer, and capsule) during their initial stages, STICs and early-stage HGSCs located on the surfaces of the fimbriated ends of the fallopian tube are directly exposed to the peritoneal cavity, allowing tumor cells to freely disseminate into the peritoneal cavity, where they may adhere to the peritoneal or mesenteric wall, resulting in the formation of multiple tumor nodules. This idea explains the diagnosis of peritoneal primary HGSCs with minimal ovarian involvement. Although the paradigm well elucidates the clinicopathological facts about the genesis of HGSC, it should not be interpreted that the fallopian tube is the exclusive origin. Many findings indicate that HGSC may occasionally develop from serous borderline tumors and low-grade serous carcinomas [31–33]. The metachronous examination of ovarian serous borderline tumors based on somatic mutations also showed the emergence of subsequent HGSCs [34].

Accordingly, there are at least two routes for HGSC development. One is by the conventional STIC route, where malignant transformation happens right on the fallopian tube before the tumor cells spread. The other is through the stepwise evolution from tubal-type ovarian serous cystadenoma and serous borderline tumors to low-grade serous carcinomas that give rise to HGSC. The neoplastic transformation in the latter pathway occurs in those fallopian tube epithelial cells within ovarian tissues [35]. Alternatively, HGSC has been reported in a serous cystadenoma on the background of an STIC lesion, suggesting that rare HGSCs can arise from serous cystadenomas [36]. Given that serous cystadenomas, serous borderline tumors, and low-grade serous carcinomas are most likely the descendants of the fallopian tube epithelium that form inclusion cysts undergoing low-grade tumor formation, the existence of HGSCs independent from STICs does not refute the validity of the tubal paradigm.

## 3. Molecular landscape changes in the pathogenesis of STIC

Investigating the primary molecular genetic occurrences is essential for elucidating the genesis of STICs from fallopian tubes. Over the past decade, several studies have reported molecular alterations in tubal precursor lesions as compared to the histologically unremarkable fallopian tube epithelium. Most of the studies report on the upregulation of specific gene products, including those involved in transcriptional regulation, cell cycle control, DNA demethylation, chromatin remodeling, DNA damage repair, long interspersed element-1 (LINE-1) demethylation and mobility, microtubule binding, cell adhesion, extracellular matrix, secreted proteins, and classical oncogenes. Gene products were downregulated in STICs as compared to the normal fallopian tube epithelium, including PAX2 and ALDH1A. Some of those studies also compared gene expression patterns in different precancer lesions, including p53 signatures and STILs, alongside STICs and HGSCs. In general, the transition from p53 signatures to STICs involves an increased number of somatic mutations, novel methylation changes, and LINE-1 activation, and the transition from STICs to HGSCs involves a further increase in somatic mutations, a higher level of aneuploidy, centrosome amplification, and more frequent immune cell infiltration.

Of great interest is the dynamic change in telomere length in precancer lesions. Telomeres shorten in STICs but do not shorten further in the transition from STICs to HGSCs [37–39]. This finding may highlight an important insight into the biology of STIC formation. Telomere attrition in the tubal epithelium may result from oxidative stress generated by follicular fluids during ovulation, leading to chromosomal instability and, subsequently, STIC development. An STIC progressing to an HGSC may have evaded telomere-induced senescence and have undergone further molecular alterations that preserve telomere length. This discovery highlights the significance of telomere stability in tumor progression. Based on nucleotide sequencing analyses, researchers find no recurrent specific somatic mutations, except *TP53* mutations, during the progression from normal-appearing fallopian tube epithelium (NFTE) to p53 signature and STIC [11, 21, 22].

Consequently, researchers have explored other pathways, such as aneuploidy, anticipating that the examination of chromosomal abnormalities in p53 signatures and STICs will provide insights into the development of ovarian cancer precursors. Aneuploidy denotes the acquisition or loss of whole chromosomes or sub-chromosomal segments. Aneuploidy arises from chromosomal instability and contributes causally to tumor growth. Conventional approaches for analyzing aneuploidy encounter technical difficulties when the target lesions are exceedingly small, such as in STICs. A technology known as the Repetitive Element Aneuploidy Sequencing System (Real-SeqS) has recently been developed. This technique utilizes a single primer pair to amplify about 350,000 amplicons of repetitive regions throughout the genome [40, 41]. According to the patterns of chromosomal abnormalities revealed by the Real-SeqS, researchers are able to distinguish morphologically defined p53 signatures, STILs, and STICs in formalin-fixed and paraffin-embedded tissues.

A recent study applying the Real-SeqS concluded that p53 signatures, the presumed earliest lesions of HGSC pathogenesis, harbored chromosome 17 loss [42]. The deletion of the entire chromosome 17 or its arm can be identified in p53 signatures, STILs, STICs, and HGSCs, while HGSCs and STICs exhibit significant aneuploidy, affecting a greater number of chromosomal locations compared to STILs and p53 signatures. In addition to TP53, several tumor suppressors, such as BRCA1, NF1, and HOXB, are located on chromosome 17.

## 4. Histopathological heterogeneity among STICs and its clinical implications

Although a comprehensive clinical correlative study is forthcoming, at least three recent small case series have indicated that STICs exhibit clinical variability, with certain STICs demonstrating clinical aggressiveness linked to subsequent disseminated HGSC [43–45]. Following risk-reduction salpingo-oophorectomy (RRSO) with incidental STIC, the probability of developing peritoneal HGSC carcinomatosis increased to 14.5% and 27.5% at the 5- and 10-year follow-ups, respectively. Despite their heightened risk, most women diagnosed with STIC following RRSO are presently not advised to undergo adjuvant chemotherapy, thereby continuing to face a significant risk of subsequently developing peritoneal HGSC [46]. However, if an incidental STIC is identified (without cancer), no consensus of care exists [47]. A critical inquiry in clinical management is whether women with incidental STICs or STILs will benefit most from chemotherapy, intensive monitoring, or only follow-up care. Thus, there is an unmet need to understand the pathogenesis that contributes to disseminated HGSC.

Recent investigations indicate that STICs consist of multiple morphologically different lesions [25, 48], even though they are presently categorized as STICs. In the most extensive cohort reported to date [48], pathologists examined 171 STICs and 21 STILs for their morphological and molecular characteristics and categorized them into two morphological subtypes: one with a flat surface (Flat) and the other displaying budding, loosely adherent, or detached (BLAD) morphology. BLAD-type lesions were more frequently indicative of STIC compared to Flat lesions and were related to concurrent HGSC. BLAD-type lesions exhibited greater proliferative activity, epithelial stratification, and more intense lymphocyte infiltration compared to Flat lesions. Notably, BLAD-type STICs exhibit an elevated Ki-67 proliferation index and exhibit DNA copy number increase or amplification at the *CCNE1* or *MYC* loci, which are also present in HGSCs.

Alongside the histopathological features and aneuploidy markers, spatial transcriptomic analysis also reveals heterogeneity in the gene expression in STICs and SITLs [49]. Compared to the adjacent histologically unremarkable fallopian tube epithelium, some of the STICs express high levels of insulin-like growth factor binding protein-2 (IGFBP2) [49]. IGFBP2 is a well-established growth factor for several types of cancer cells, including ovarian carcinomas, through multiple mechanisms, such as reprogramming cancer cell metabolism. Immunohistochemistry confirmed this discovery and showed that most STICs, but not STILs or p53 signatures, had IGFBP2 immunoreactivity, but the intensity and percentage of positive STIC cells varied among them. IGFBP2 expression is associated with BLAD morphology and the high proliferative activity of STICs. A further study has shown that demethylation of the proximal enhancer of the IGFBP2 gene increases its protein expression and sustains the proliferation in STIC lesions.

Since long-term follow-up information is unavailable in most of these STIC cases, whether BLAD lesions, amplification of *CCNE1*, and overexpression of IGFBP2 are associated with an increased risk for subsequently developing disseminated HGSC remains uncertain. Nevertheless, the morphological and molecular markers provide a foundation for future clinical correlation.

## 5. STIC-like lesions with concurrent high-grade serous carcinoma

**Figure 3 •:**
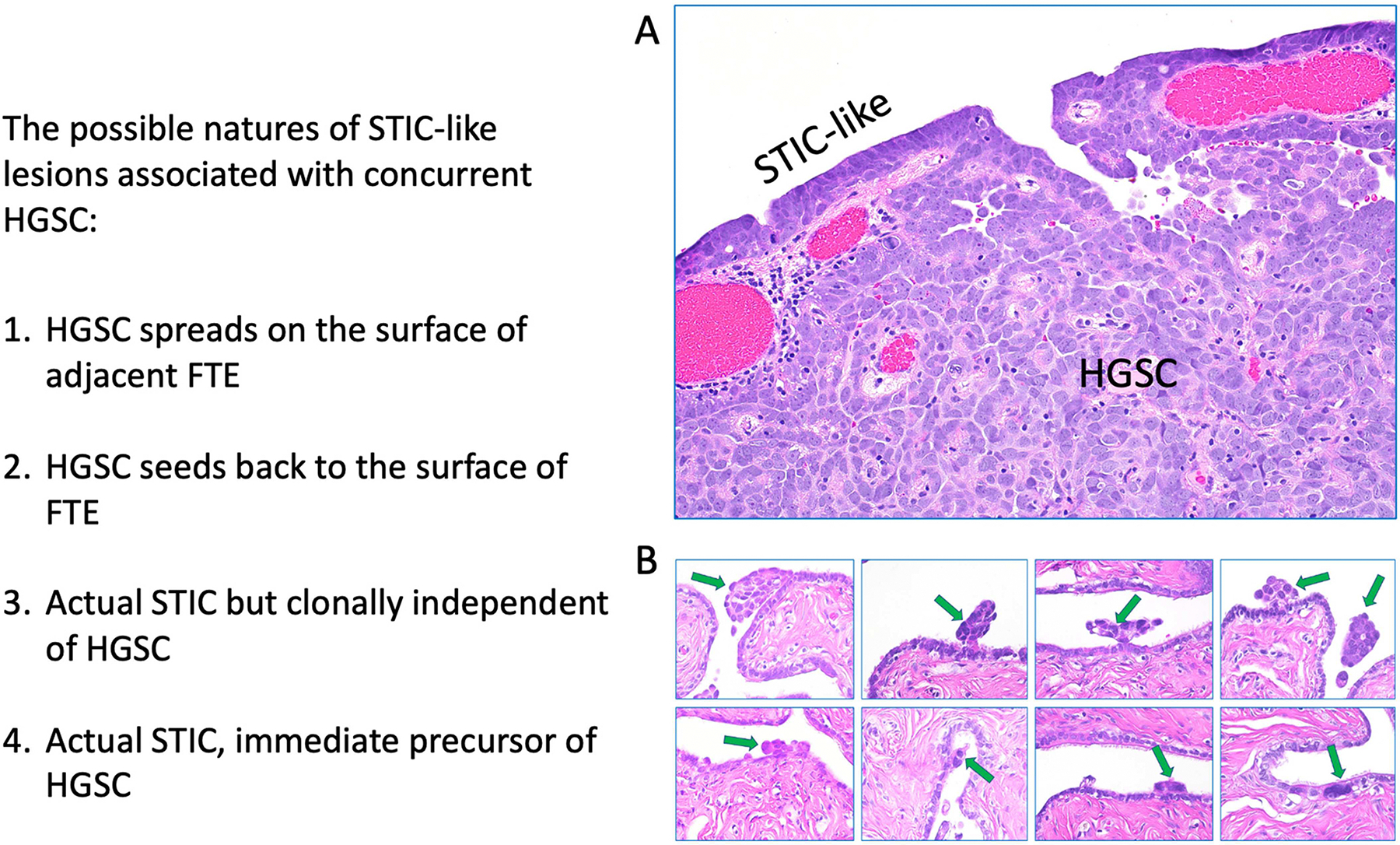
The possible natures of “STIC-like” lesions in association with HGSCs. There are at least four explanations for the presence of “STIC-like” lesions resembling isolated STICs. (**A**) An example likely representing the first possibility. The HGSC cells efface and replace FTE, assuming the appearance of STICs. (**B**) Examples of the seeding back theory that isolated HGSC clusters in the ascites may adhere to the surface of FTE, proliferate, and lateral spread with the tubal surface, mimicking STICs. Those STICs, especially those that are geographically away from HGSCs, may be the actual STICs. Most likely, they are the genetic “cousins” of the HGSCs and are not immediate precursors of HGSCs. Rarely, those lesions may genuinely represent the residual precursor for the concurrent HGSCs. Green arrows indicate the “floating” fragments of HGSC cells adhering to the surface of fallopian tube mucosa.

One of the most pressing concerns in the molecular characterization of STIC is the comparison of “STIC” lesions with nearby HGSC tissues from the same patients. This method is justifiable since such analyses have traditionally provided essential insights into the development from a precursor to an invasive carcinoma. Anecdotal evidence suggests that “STICs” associated with HGSCs may indicate the “seeding back” of peritoneal HGSC cells, resembling de novo STICs (Figure 3). Consequently, the proximity of an HGSC and an STIC does not necessarily mean that the HGSC originated directly from the neighboring STIC. Prior evolutionary trajectory analysis utilizing nucleotide sequence mutations as markers indicates that HGSCs can be traced to specific STICs. However, those studies were based on using formalin-fixed and paraffin-embedded tissues whose DNA quality and quantity may introduce sequence noise that leads to miscalls of somatic mutations, confounding the conclusions that STICs were the precursors of some HGSCs in those samples.

The existence of STICs in HGSC patients carries multiple biological ramifications. Primarily, it is exceedingly challenging to rationalize that the STIC is the precursor lesion of the associated HGSC. This occurs because, in that situation, the minute precursor lesion, the epicenter of HGSC growth, has been supplanted or obliterated by bulk HGSC cells, which outnumber STIC by millions. The mechanism by which the extensive HGSC tissue selectively preserves its ancestral precursor after a significant temporal interval is still conjectural. To acknowledge this potential complication in the molecular studies of STIC, we designate these lesions as “STIC-like” in instances of concurrent HGSC. This aims to differentiate “STIC-like” lesions from incidental or isolated STICs, which probably signify the actual precursor lesions. These incidental STICs may be considered the most pristine precursor lesions for investigating molecular etiology prior to the onset of cancer. Conversely, the existing STIC-like lesions that persisted throughout HGSC advancement are authentic STICs; nevertheless, they may originate from distinct regions of the fallopian tubes not affected by HGSC and do not inherently represent the precursor of HGSC.

## 6. Genetic predisposition to ovarian cancer in *BRCA1* germline mutations

A primary conclusion derived from several molecular genetic studies is that *TP53* mutation and chromosomal 17 loss represent two almost universal events in initiating the transformation of tubal epithelium (p53 signatures). As *TP53* and *BRCA1* reside on chromosome 17, the deletion of this chromosome simultaneously eliminates both tumor suppressor genes in the same allele. Regarding the risk of ovarian cancer, women are typically categorized into two groups. The first group consists of “high-risk” women due to inherited pathogenic mutations (predominantly *BRCA1*, *BRCA2*, *RAD51C*, *RAD51D*, and *PABL2*) and/or familial histories of ovarian and breast cancer. The other group consists of those at “average risk” lacking these supplementary risk factors [50, 51]. Since the fallopian tube epithelium of BRCA1 mutation carriers possesses a germline inactivating *BRCA1* mutation, all the p53 signatures in *BRCA1* mutation carriers harbor “two hits” to both *TP53* and *BRCA1*. This mechanism may help clarify why those high-risk women, as compared to average-risk women, are 20 to 35 times more likely to develop HGSC during their lives, frequently at younger ages.

In principle, since all p53 signatures exhibit *TP53* missense mutations and demonstrate the entire deletion of one chromosome 17, epithelial cells inside p53 signatures may have an increased propensity to progress into STICs in accordance with the “double hit” hypothesis. These ostensibly normal fallopian tube epithelial cells exhibiting p53 signatures had two alterations in *TP53*: one allele has a gene deletion, while the other contains a missense mutation. Secondly, the p53 signature epithelium in non-*BRCA1* women may theoretically resemble the NFTE resulting from a germline BRCA1-inactivating mutation; as in both scenarios, one *BRCA1* allele remains intact while the other is inactivated or deleted. Conversely, it is probable that the p53 signature epithelium in *BRCA1* carriers exhibits multiple hits in both *TP53* and *BRCA1* due to somatic mutations on one chromosome and the concurrent deletion of the other.

Accordingly, we propose that fallopian tubes harboring *BRCA1* detrimental germline mutations have a hereditary predisposition for p53 signatures to STIC. This offers a plausible rationale for the increased risk and earlier onset of HGSC associated with *BRCA1* germline mutations. Moreover, the *TP53* mutation is recognized as the first and most critical event in the development of HGSC. Despite the bi-allelic mutations in *TP53* among non-*BRCA1* carriers and the presence of both *TP53* and *BRCA1* mutations in *BRCA1* carriers, mathematical modeling and epidemiological studies suggest that the progression to an STIC may take several decades. Once an STIC develops, it often progresses to invasive HGSC within an additional 6 to 7 years.

## 7. Potential promises and challenges in studying STICs

There are several promises stemming from this new tubal paradigm. First, as the paradigm implies, salpingectomy can be preventive for HGSCs. In fact, three population-based studies support this view [23, 26, 27]. Opportunistic salpingectomy is a procedure to reduce ovarian cancer risk by giving an opportunity to assess the fallopian tubes during an indicated intra-abdominal surgery. In recent years, opportunistic salpingectomy has gained increasing attention as a primary prevention of HGSC in the general population [26, 52–54]. Based on a systematic literature review, a recent study reports that bilateral salpingectomy was a clinically feasible, safe, and potentially cost-saving procedure for women [54]. From this perspective, surgical pathologists will encounter an increasing number of such cases. They are encouraged to report “opportunistic salpingectomy” in their pathology reports for documentation and for future research purposes.

Second, the paradigm offers a unique model for studying the pathogenesis in HGSC initiation. Table 1 summarizes some of their key findings known so far. Elucidating the biology of STICs serves as a foundation to discover biomarkers for early diagnosis. For instance, the ongoing development of the STIC multi-omics atlas will likely provide multi-dimensional data and integrated analyses to elucidate how molecular genetic alterations, epigenetic reprogramming, and pathway reconfiguration contribute to the emergence of the transformed tubal epithelial cells and the onset of the earliest tubal lesions. Utilizing publicly available datasets, researchers will enter into a new horizon to study the pathogenesis of HGSC in the near future. Gynecologists will investigate the clinical importance of isolated STICs and inquire if it is feasible to risk-stratify STICs in those samples from risk-reduction and opportunistic salpingectomy. By correlating histopathological and genetic findings, pathologists can standardize their diagnostic criteria in defining STICs. Furthermore, computational biologists are well equipped to illustrate the evolutionary history and timing of tumor start and progression in STIC and STIL. Similarly, the STIC atlas will assist molecular biologists in identifying STIC-associated genes and their corresponding targetable pathways to formulate non-surgical preventative measures for high-risk women seeking to postpone salpingectomy, if needed. Epidemiologists and biologists will ascertain whether the early diagnosis of STICs and incipient HGSC can preserve women’s lives by utilizing liquid Pap cervical samples or fluid from uterine lavage to identify STIC- or HGSC-related markers, including protein and methylation markers [12, 55, 56].

Notwithstanding the aforementioned promises, several challenges are also acknowledged. Foremost, a lack of longitudinal follow-up data with sufficient time on women diagnosed with incidental STICs languishes in all efforts to learn the clinical significance of morphological and molecular changes. We propose a nationwide STIC consortium that coordinates STIC reporting, collection, pathology diagnosis, and tissue sharing from participating institutions or from patients. Significant progress has been achieved through the spatial analysis of omics, even at the single-cell level, on those minute formalin-fixed and paraffin-embedded STICs and STILs. Multiplex immunostaining with *in situ* hybridization also provides a practical and convenient method to validate the markers from omics studies. However, innovative research can only become meaningful if the correct lesions are identified and analyzed in the very beginning. Without active involvement by pathologists, the research data and conclusions may be jeopardized. The above promises and challenges highlight the essential involvement of multi-disciplinary teams.

## 8. Conclusions

**Figure 4 •:**
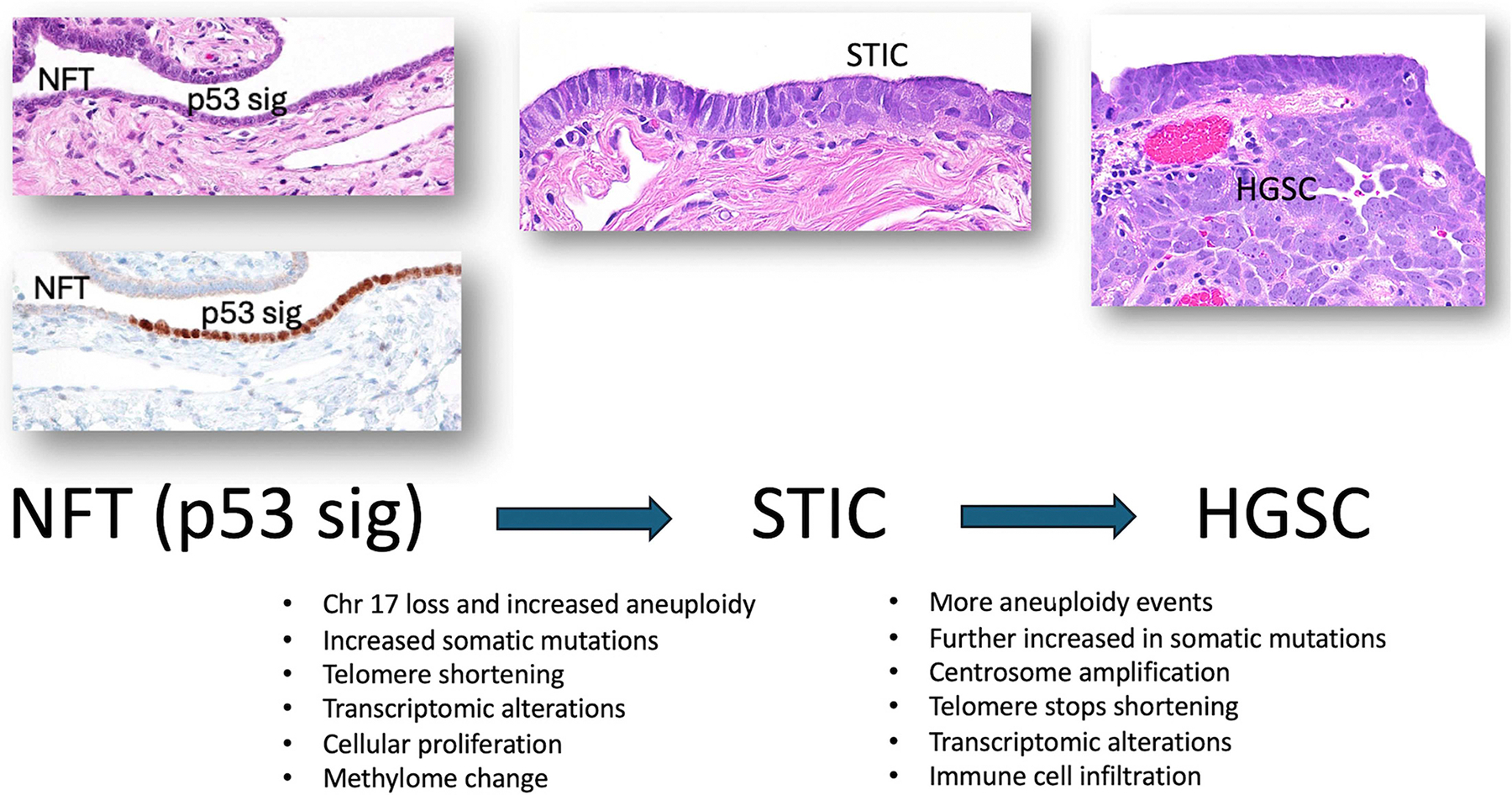
Summary of molecular changes in transition between normal fallopian tube (NFT) epithelium and p53 signature to serous tubal intraepithelial carcinoma (STIC) and between STIC to high-grade serous carcinoma (HGSC). Those molecular alterations indicate an increasing genomic instability during tumor initiation to HGSC, as evidenced by increasing aneuploidy, somatic mutation number, and associated transcriptomic and epigenetic changes. Immune cell infiltrate is more often observed in HGSCs than STICs.

Since the tubal paradigm of HGSC was born two decades ago, we have witnessed burgeoning research activity in describing tubal precursor lesions by pathologists and basic researchers who characterize the morphological and molecular alterations unique to p53 signatures, STILs, and STICs. Several conclusions can be made from those studies. First, *TP53* mutations and loss of chromosome 17 represent the earliest molecular genetic events known so far in initiating HGSC, as the alterations can be detected in p53 signatures. Second, the co-localization of *TP53* and *BRCA1* genes in chromosome 17 may explain the genetic predisposition of HGSC in women carrying BRCA1 germline pathogenic mutations. Third, an increasing level of nuclear atypia, chromosomal instability, somatic mutation burden, and selected dysregulated genes are associated with the progression from normal fallopian tube epithelium to p53 signatures, STILs, STICs, and to HGSCs (summarized in Figure 4). Fourth, the tubal paradigm transforms multiple facets of early diagnosis and detection of HGSCs, including their precursors. Opportunistic salpingectomy likely has a clinical benefit for the surgical prevention of HGSC in average-risk women. Multi-omics investigations aimed at identifying STIC- and HGSC-related tissue biomarkers promise potential for early detection and diagnosis using standard liquid-based cervical Pap tests. More collaborative efforts, like organizing national STIC consortia to collect specimens and follow-up data, will likely provide information as to whether some women with incidental STICs are more likely to develop subsequent disseminated HGSC.

## Figures and Tables

**Table 1 •:** Examples of molecular changes and pathways they involved in the development of STICs.

Alteration types	Gene symbol	Gene name	Function	Main findings	References
Mutations and LOH	*TP53*	Tumor protein p53	It responds to cellular stresses to regulate the expression of target genes, thereby inducing cell cycle arrest, apoptosis, senescence, DNA repair, or changes in metabolism.	Mutated in p53 signature, STIL, STIC and HGSCLOH in STIC and HGSC	[57]
Mutations	*NF1*	Neurofibromin 1	Negative regulator of the ras signal transduction pathway.	Mutated in STIC	[11]
Change in expression	P16 (*CDKN2A*)	Cyclin-dependent kinase inhibitor 2A	Inhibits CDK4 kinase and stabilizes p53, thereby involving cell cycle G1 control.	Overexpressed in STIC and HGSC	[18, 58]
Change in expression	Rsf-1 (*HBXAP*)	Remodeling and spacing factor 1(hepatitis B X-antigen-associated protein)	Involved in chromatin remodeling and facilitates transcription of hepatitis B virus genes by the HBX transcription activator.	Increased expression in STIC and HGSC	[58]
Change in expression	*FASN*	Fatty acid synthase	Catalyzes the synthesis of palmitate from acetyl-CoA and malonyl-CoA into long-chain saturated fatty acids.	Increased expression in STIC	[58]
Change in expression	*MUC4*	Mucin-4	Encodes an integral membrane glycoprotein found on the cell surface; secreted isoforms may exist.	Decreased expression in STIC	[58]
Change in expression	*Ki67*	Antigen identified by monoclonal antibody Ki 67	Involved in regulation of chromatin organization.	Ki67 labeling index is elevated (> or = 10%) in STIC	[58]
Change in expression	*LAMC1*	Laminin γ1	Enables glycosphingolipid binding activity; acts upstream of or within neuron projection development; located in basement membrane and synaptic cleft.	Increased expression in STIC and HGSC	[59]
Amplification and change in expression	*CCNE1*	Cyclin E1	Functions as a regulatory subunit of CDK2, whose activity is required for cell cycle G1/S transition. Overexpression of this gene has been observed in many tumors, which results in chromosome instability.	Positive expression in STIC and HGSC Amplified in STIC and HGSC	[58, 60]
Change in expression	*TET1*	Ten–eleven translocation methylcytosine dioxygenase-1	DNA demethylation	Overexpressed in STIC and HGSC	[61]
Change in expression	*PAX2*	Paired box 2	Involved in negative regulation of apoptotic process involved in development; nervous system development; and urogenital system development.	Expression reduced in STIC	[62]
Change in expression	*STMN1*	Stathmin 1	Involved in the regulation of the microtubule filament system by destabilizing microtubules; prevents assembly and promotes disassembly of microtubules.	Overexpressed in STIC	[18]
Change in expression	*L1CAM*	L1 cell adhesion molecule	Involved in nervous system development, including neuronal migration and differentiation.	Overexpressed in STIC	[63]
Change in expression	*Ki67*	Antigen identified by monoclonal antibody Ki 67	Involved in regulation of chromatin organization.	Ki67 labeling index is elevated (> or = 10%) in STIC	[58]
Change in expression	*LAMC1*	Laminin γ1	Enables glycosphingolipid binding activity; acts upstream of or within neuron projection development; located in basement membrane and synaptic cleft.	Increased expression in STIC and HGSC	[59]
Amplification and change in expression	*CCNE1*	Cyclin E1	Functions as a regulatory subunit of CDK2, whose activity is required for cell cycle Gi/S transition. Overexpression of this gene has been observed in many tumors, which results in chromosome instability.	Positive expression in STIC and HGSC Amplified in STIC and HGSC	[58, 60]
Change in expression	*TET1*	Ten-eleven translocation methylcytosine dioxygenase-i	DNA demethylation	Overexpressed in STIC and HGSC	[61]
Change in expression	*PAX2*	Paired box 2	Involved in negative regulation of apoptotic process involved in development; nervous system development; and urogenital system development.	Expression reduced in STIC	[62]
Change in expression	*STMN1*	Stathmin 1	Involved in the regulation of the microtubule filament system by destabilizing microtubules; prevents assembly and promotes disassembly of microtubules.	Overexpressed in STIC	[18]
Change in expression	*L1CAM*	Li cell adhesion molecule	Involved in nervous system development, including neuronal migration and differentiation.	Overexpressed in STIC	[63]
Change in DNA methylation and expression	*L1ORF1p*	LINE-1 open reading frame 1 protein	Binds and protects LINE-1 RNA and is required for LINE-1 mobility.	Promoter hypomethylation and overexpression in STICOverex-pressed in HGSC	[64, 65]
Telomere	NA	NA	A region of repetitive DNA sequences at the end of a chromosome; protect the ends of chromosomes from becoming frayed or tangled.	Shortened in p53 signatures and STIC	[37, 39]
Change in DNA methylation and expression	*IGFBP2*	Insulin-like growth factor binding protein 2	Binds IGF-I and IGF-II in blood, or remains intracellular, interacting with many different ligands. High expression levels of this protein promote the growth of several types of tumors.	DNA hypomethylation and overexpression in STIC and HGSC	[66]
DNA methylation	NA	NA	An epigenetic mechanism that regulates gene expression and tissue differentiation	STIC and HGSC share regions of differential hypermethylation	[12]
Aneuploidy	NA	Chri7 aneuploidy	Contains genes commonly altered in HGSC, including TP53, BRCA1, NF1, and HOXB.	Occurs in all precursor lesions and HGSC	[48, 60, 66]
Aneuploidy	NA	Chr22q loss	NA	Occurs in majority of STIL, STIC, and HGSC	
Aneuploidy	NA	Focal amplification of 19q12 or 19q13.2	Contains genes commonly altered in HGSC, including CCNE1, EIF3G, DNMT1, CDKN2D.	Mainly occurs in STIC and HGSC	
Aneuploidy	NA	Chr8q gain or focal 8q24 gain	Contains genes commonly altered in HGSC, including MYC, RECQL4, SOX17, MARK15, FOXH1, GLI4, SHARPIN, HSF1.	Mainly occurs in STIC and HGSC	
Change in expression	*ALDH1A1*	Aldehyde dehydrogenase 1 family member A1	Involved in alcohol, high-fat diet, and retinol metabolism.	Expression reduced in P53 signature and STIC; low expression in HGSC	[67, 68]
Change in expression	*PTEN*	Phosphatase and tensin homolog	Negatively regulates intracellular levels of phosphatidylinositol-3,4,5-trisphosphate in cells and functions as a tumor suppressor by negatively regulating AKT/PKB pathway.	Expression reduced in STIC and HGSC	[62]
Loss of heterozygosity	*BRCA1*	BRCA1 DNA repair associated	Involved in maintenance of genome stability, specifically the homologous recombination pathway for double-strand DNA repair.	Occurs in STIC and HGSC	[11]
Loss of heterozygosity	*BRCA2*	BRCA2 DNA repair associated	Involved in maintenance of genome stability, specifically the homologous recombination pathway for double-strand DNA repair.	Occurs in STIC and HGSC	[11]
Loss of heterozygosity	*RB1*	RB transcriptional corepressor 1	Negative regulator of the cell cycle; stabilizes constitutive heterochromatin to maintain the overall chromatin structure; binds transcription factor E2F1.	Occurs in STIC and HGSC	[11]

NA: gene name not available as the molecular changes involve chromosomal structures.

## Data Availability

Data supporting these findings are available within the article, at https://doi.org/10.20935/AcadOnco7620, or upon request.
